# Facing famine: Somali experiences in the famine of 2011

**DOI:** 10.1016/j.foodpol.2016.11.001

**Published:** 2016-12

**Authors:** Daniel Maxwell, Nisar Majid, Guhad Adan, Khalif Abdirahman, Jeeyon Janet Kim

**Affiliations:** Feinstein International Center, Friedman School of Nutrition Science and Policy, Tufts University, 114 Curtis Street, Somerville, MA 02144, USA

**Keywords:** Famine, Somalia, Coping, Social connectedness, Social exclusion, Humanitarian assistance

## Abstract

In 2011–12, Somalia experienced the worst famine of the twenty- first century. Since then, research on the famine has focused almost exclusively on the external response, the reasons for the delay in the international response, and the implications for international humanitarian action in the context of the “global war on terror.” This paper focuses on the internal, Somali response to the famine. Themes of diversification, mobility and flexibility are all important to understanding how people coped with the famine, but this paper focuses on the factor that seemed to determine whether and how well people survived the famine: social connectedness, the extent of the social networks of affected populations, and the ability of these networks to mobilize resources. These factors ultimately determined how well people coped with the famine. The nature of reciprocity, the resources available within people’s networks, and the collective risks and hazards faced within networks, all determined people’s individual and household outcomes in the famine and are related to the social structures and social hierarchies within Somali society. But these networks had a distinctly negative side as well—social identity and social networks were also exploited to trap humanitarian assistance, turn displaced people into “aid bait,” and to a large degree, determined who benefited from aid once it started to flow. This paper addresses several questions: How did Somali communities and households cope with the famine of 2011 in the absence of any state-led response—and a significant delay in a major international response? What can be learned from these practices to improve our understanding of famine, and of mitigation, response and building resilience to future crises?

## Highlights

• Somalia experienced the worst famine of the twenty-first century in 2011.
• People’s ability to cope with the famine depended on diversification, mobility and flexibility.
• People’s social connections were instrumental in how well they coped with the famine.
• Social networks functioned differently in famine—and were not always able to sustain members.
• Understanding social connectedness will improve famine prevention and response.

## 1 Introduction

In 2011–12, Somalia experienced the worst famine of the twenty-first century. Since then, research on the famine has focused almost exclusively on the *external* response, the reasons for the delay in the international response, and the implications for international humanitarian action in the context of the “global war on terror.” This paper focuses on the *internal*, Somali response to the famine. How did Somali communities and households cope with the famine of 2011 in the absence of any state-led response—and a significant delay in a major international response? What can be learned from these practices to improve our understanding of famine, and of mitigation, response and building resilience to future crises?

Themes of diversification, mobility and flexibility are important in understanding how people coped, but this paper focuses on the factor that most determined how well people survived the famine: social connectedness or “capital.” The nature of reciprocity, and especially the resources available within people’s networks, and the collective risks and hazards faced within networks, all determined people’s individual and household outcomes in the famine. Many of these factors and dynamics are related to the social structures and social hierarchies within Somali society.

This paper briefly reviews the literature on famine “coping strategies” and on “social capital.” Then it presents a synthesis of evidence from research on the Somali famine of 2011 that apply to a range of coping practices, highlighting the ways in which social connections enabled people to survive, but also put certain groups of people at much greater risk. Finally, the paper suggests several working hypotheses incorporating the implications for theory, policy, and practice.^1^

### 1.1 Methodology

This paper is based on 260 narrative interviews on the famine from the perspective of people most affected by it.^2^ These come from interviews conducted in 2012–14 in Bay, Lower Shabelle, Gedo, Middle Juba, Galgadud and Mudug regions, as well as in Mogadishu, and in the refugee camps in Dollo-Ado, Ethiopia and Dadaab, Kenya. A small number of additional interviews were also conducted in Nairobi in the UK, with populations sending remittances.^3^ The interviews noted clan identities and in all locations other than displaced and refugee camps, purposively selected two or three of the different clan or social groups in the local research site.^4^ This analysis was informed by a grounded theory approach,^5^ but the interviews were framed in light of the anthropological literature on Somali society. Nevertheless, the interview questions evolved over time and by location during the study, so direct quantitative comparisons or counting of emerging thematic trends is not possible from this evidence. Conclusions here are framed as working hypotheses about the nature of coping and social relations in Somali society.

Conducting interviews with a sensitivity to social identity allowed the analysis to differentiate trends in coping and survival strategies among different social groups, including how commonly remittances were received (from within the country and abroad), how people described the way in which they called on relatives, clan-members, or others in distant locations, how important having relatives in urban areas or abroad was as a livelihood or survival factor. Analysis of patterns and differences within the same area as well as across the whole sample population revealed the major findings in this paper. This paper uses quotes from specific individuals to illustrate these wider patterns and indicate how some groups and/or households are very strongly connected between rural, urban and diasporic locations; the extent of this phenomenon is such that even respondents and who are not connected in this way highlight a significant coping and survival strategy for many people.

Somali social structure is highly complex and variable.^6^ As Roland Marchal puts it, “history, migration and urbanisation … have made each clan very distinct, even while they claim many commonalities.”^7^ However, lineage identity is a powerful form of social solidarity, that includes sharing of resources, and is commonly invoked through notions such as *diya* and *qaraan*—the latter as a collective form of insurance payment in relation to conflict, the latter as a more general intra-clan fund-raising system.^8^ It also provides the structure through which relationships can be identified and thereby assistance requested. That said, others forms of relationship that can generate assistance also exist, such as friendship, business relations and religious affiliations. To the extent possible, all these processes and variations of the Somali social structure—as they functioned in a crisis of the severity of 2011—were queried as components of interviews. Evidence from the interviews and multiple rounds of analysis and triangulation with Somalis and Somali experts provide sufficient confidence that working hypotheses regarding Somali social networks can be suggested.

While the importance of clan identity and its social connectedness came out as a significant factor in relation to social solidarity and survival, it also provided the separation of identity that allowed weaker groups, particularly the displaced who were predominantly the Rahanweyn and Jarer, to be preyed upon by more powerful groups, into whose area they were moving.

### 1.2 Background on the famine

The famine itself, which has been well documented elsewhere,^9^ resulted in the loss of an estimated 258,000 human lives.^10^ It was triggered by drought and a major production failure, by a global spike in the price of food that drastically reduced people’s purchasing power at a time when local production had failed, and by an on-going war. The lack of adequate preventive measures was at least in part because a proscribed group controlled much of the affected area, and counter-terrorism legal restrictions outweighed humanitarian concerns in external policy consideration.^11^ Both the controlling local authority (Al-Shabaab) and international donors put severe restrictions on humanitarian action that could have prevented or mitigated the crisis—and significantly delayed a major international response. As a result, many affected groups were forced to deal with the worsening crisis almost entirely using their own mechanisms and social networks.

A large-scale crisis had been predicted as early as late-2010, but not enough was done to mitigate the onset of the disaster. The evidence suggests that the worst of the mortality had already peaked by the time that the famine was declared.^12^ The declaration mobilized a large-scale response by both Western and non-Western humanitarian actors (primarily from Islamic donors and charities), and combined with the return of the rains and a rapid reversal in the high cost of food, brought the remaining mortality under control by early 2012.^13^ But the major response was very late and the factors just outlined prevented the response from reaching some of the worst affected areas. Before the major international response (by both Western and non-Western actors)^14^ reached affected groups, they drew support from their own communities, their business groups, their Diasporas, and their own neighbors and kin. This paper briefly analyses these responses.

## 2 Coping and social connectedness

### 2.1 Coping with famine and acute food insecurity

Research on coping with food security crisis can be traced back to Amartya Sen’s seminal work on “entitlements.”^15^ Sen’s suggestion that the process of entitlement failure could be mapped led to considerable research on “coping strategies,” summarized by Corbett in 1988.^16^ Michael Watts suggested that, as food access becomes more constrained, households are more likely to employ less reversible and more severe strategies, attempting to reduce short-term threats to food access while maintaining the longer-term viability of livelihoods.^17^

Other research in the area of coping and adaptation includes work on the *intensification* of existing strategies, the *diversification* of activities and *migration* in search of new opportunities.^18^ Migration has long been observed as a livelihood option in both the short term and the long term, depending on labor demand between different areas.^19^ As the costs of telecommunications and transportation have declined, the connections between local communities and distant kin have increased. In some cases, transnational social networks are such that people in different countries can be described as effectively being part of the same “community.”^20^ Remittance income has been repeatedly shown to be a small but significant factor in the incomes of poor rural households. Migration to urban areas, and to destinations outside of the country of origin are also a prominent features of livelihood strategies.^21^ But coping with crisis also clearly depends on the social networks or the social connectedness of affected households and communities.

### 2.2 Social “capital” and social connectedness

Three major sources of empirical work helped to define social capital.^22^ Bourdieu noted that social capital is “an aggregate of the actual or potential resources which are linked to possession of a durable network of a more or less institutionalized relationships or mutual acquaintance or recognition”^23^—in other words, the network of social ties that a person could access and the resources that could then flow through them. Three forms of capital existed at the individual level and were dependent of an individual’s social attributes, his/her capacity to accumulate these types of capital, and the relevance of these capital to the individual’s context. Coleman on the other hand, asserted that the definition of social capital lies more in the function it is able to perform: “It is not a single entity but a variety of different entities, with two elements in common: they all consist of some aspect of social structure, and they facilitate certain actions of actors… within the structure.”^24^ For Coleman, social capital was a public good, not just confined to be an individual resource. Putnam built on Coleman’s approach to emphasize the public good nature of social capital and defined it as a community-level attribute. According to Putnam, “social capital is the ‘feature of social organization, such as trust, norms, and networks, that can improve the efficiency of society by facilitating coordination actions.’”^25^

Social capital has frequently been broken down into three dimensions.^26^ *Bonding* social capital refers to bonds between people to whom Putnam refers as “homogenous” community members and involves principles and norms such as trust, reciprocity, and cooperation.^27^ These horizontal ties exist between similar individuals resulting in a strong sense of belonging—to a group, tribal unit, or nation. They can also represent indifference or even hostility, escalating to deliberate polarization, isolation, or even violence towards non-members.^28^ *Bridging* social capital links members of one group to another across, for example, ethnic or racial lines, geographic boundaries, and language groups.^29^ In turn, bridging social capital helps foster connections to *external* assets and different social or economic identities. These linkages can foster community resilience, drawing on them when local resources are depleted or scarce. Finally, *linking* social capital refers to “networks of trusting relationships between people who are interacting across explicit, formal, or institutionalized power or authority gradients in society.”^30^ Bonding and bridging social capital usually refer to connections between individuals of similar status; linking social capital, on the other hand, takes into account the “vertical distance” of individuals’ varying positions of authority.^31^

These forms of social “capital” have been cast as central factors in the ability to respond to shocks; they have been identified as a vital component of risk-smoothing and risk-sharing practices to help individuals, households, and communities react to and recover from disasters.^32^ Social capital is frequently presented as a public good—a resource that provides non-excludable benefits to those in the group. However, numerous examples show that social capital may be both a public and quasi-private good; that is to say, benefits do not affect individuals and/or groups in the same way.^33^ Benefits may be redeemed at the *expense* of outsiders. Indeed, gender, wealth, and other power relations shape social networks.^34^ Furthermore, these ties are not static over time but change through people’s lives and between generations, and may fade as well as be renewed.^35^

Several issues arise from the existing research that are worth noting. First, the notion of social “capital” suggests something directly fungible that could be counted, saved up, or called in; economic rather than social aspects are usually emphasized. Second, the tendency is to emphasize the positive aspects of social connections or social capital. Much of the literature ignores the way in which “social capital” can also be used for exclusionary or exploitative purposes, and downplays the extent to which trust can break down and potential support from social networks can fluctuate over time. Third, the literature on coping strategies notes specific behaviors such as borrowing money or food, or purchasing food on credit—which obviously depend on the social linkages of the household. Indeed, migration strategies, labor-sharing arrangements and risk sharing groups (such as funeral societies) all depend on the social connections of households and individuals. But beyond that, little detail has been noted on the specific linkages between social connectedness and coping. And fourth, while the literature on coping notes a hierarchy of behaviors that implies increasing severity (even while noting the context specificity of such hierarchies), there is little research on the extent to which the nature of connectedness may vary between “normal” times and times of increasing hardship. The research outlined below notes how this can work both ways; in hard times, social connections may be strengthened, but the functions of social networks may also break down.

### 2.3 Lineage, social relations and famine

The single group that suffered the highest mortality in the 2011 famine was segregated by age, not social identity *per se*,^10^ with children under the age of five being the worst affected. However, the evidence is that the highest mortality from the famine was among specific social groups, namely the Rahanweyn and the Bantu/Jarer—the same groups as were hit hardest in the famine of 1991/92 (Majid and McDowell, 2012). Research for this paper was therefore designed with sensitivity to the potential for significantly different impact between different social groups or clans. Somali society is described as a “segmented lineage” structure that subdivides along its constituent branches from generation to generation, and claims a “total Somali genealogy.”^36^

As a resource sharing structure, Gardner and El Bushra describe an extended family with members in rural, urban and diaspora locations where income and other resources are shared across long distances to maintain the integrity of the whole, extended family.^37^ However, this structure and its internal coherence as a resource sharing system arguably represents the pastoral, “noble” clans most closely (and can vary according to internal, social dynamics). The Rahanweyn clan family have a lower social status than the ‘noble’ clans and have an agro-pastoral influenced livelihood and culture (although on a spectrum from more pastoral to more sedentary farming). The Somali Bantu or Jarer, are located outside the segmentary lineage structure, and where the Rahanweyn have been described as second class citizens the Jarer are described as third class citizens.^38^

This hierarchy in Somali society as well as the diversity of resource options is embodied in the Somali diaspora. One in six Somalis was estimated to be located in the diaspora over ten years ago, with financial remittances accounting for the largest share of the economy.^39^ The “noble” clans are predominant amongst the diaspora for historical reasons. The evidence of the impact of remittances on food security in the face of extreme crises has been limited, and in any case, the role of diaspora remittances only partially captures of the notion of social connectedness.

## 3 Coping with crisis and famine in Somalia

### 3.1 A typology of coping in Somalia

The term “coping” has come to mean many things: “Coping” refers to relatively short-term changes in behavior to deal with a setback—with varying degrees of reversibility in the individual strategies employed; “adapting” refers to longer-term changes to deal with a permanently changed context.^40^ These terms correspond to categories in the contemporary literature on *resilience*: “Absorptive capacity” is about dealing with short-term setbacks and “bouncing back” (“coping” in earlier parlance). “Adaptive capacity” is about dealing with longer-term changes, while protecting future capacity (“adapting” in earlier parlance). A third capacity, “transformative capacity” is about being able to proactively shape that context.^41^

Table 1 depicts a typology of “coping strategies” that respondents described in response to the famine of 2011. These can be classified as strategies related to *diversification* (in terms of livelihood strategies or assets, but more importantly diversification of risk or exposure to hazards); *flexibility* (which includes mobility in the case of livestock-dependent livelihoods; but also the ability to exploit various opportunities); and *social connectedness* (the strength of an individual or household’s social networks and the ability to call upon others to help in the event of a shock). How strategies play out, the order in which they are invoked, and their long-term consequences vary by social group and geography.

These categories (diversification, flexibility, and connectedness) also have gender dimensions. Changing roles of both women and men, following the political and economic volatility of the last two to three decades in Somalia, have led to women becoming more active in economic pursuits. This is in part as a result of their dual identities as wives and daughters (belonging to different families through birth and marriage), which enables them to cross clan divides for political, social and economic purposes. 42 It is also due to the necessity of earning income for the family. Women’s financial contributions as remitters from the diaspora are contributing to changing perceptions of gender roles.^43^ While women traditionally had less mobility, very few women respondents reported limited mobility as a constraint to coping with the famine of 2011. Somalia remains a patriarchal society, but gender relations are changing and are more complicated than often suggested.

### 3.2 Absorptive strategies and the role of social connectedness

Strategies noted in Table 1 are, for the most part, not unique to Somalia. Most of them have been noted previously in highly risk-prone areas of the Horn of Africa and elsewhere.^44^ However, in the Somali context, social connectedness plays a particularly important role. For much of Somali society, these networks are best understood in terms of the norms of reciprocity and obligation that exist as part of belonging to a particular clan-based identity group (though agricultural labor groups, friends and other categories of connection may also be important).

*Having “someone to cry to.”* A wide variety of respondents across all the study areas used the language of having “someone to cry to” to refer to requesting assistance from someone else, where the person to whom one could cry would typically, but not exclusively, be a relative—hopefully one based in town or in the diaspora (therefore outside the immediately-affected rural economy). This general observation is illustrated by the a quote from a Rahanweyn-Leysan pastoralist:^45^ *“People who have nobody to cry to, that is who don’t have kinsmen to help, don’t have a son or a daughter in the towns or out of the country to help*… *all such people have no coping capacity. From our case, we were also affected very much in our village but the number of people who died were not many.”*^46^

Another quote comes from a displaced person in the Dollo Ado refugee camp in Ethiopia, who stated that, *“We had nobody to cry to for any form of assistance and/or remittance.”*

### 3.3 Remittances and social linkages: three circles of social obligation

The value of having “someone to cry to”—having a social network, particularly with members *outside* the rural economy—is reinforced by the extent of the Somali diaspora. However, while the strength of Somali social networks and a culture of sharing is renowned, and is applicable to all Somalis, the quality of social connectedness, in terms of links between rural, urban and diaspora locations and the availability of resources at these different locations, is markedly different between different groups. In particular, those social groups most dependent on the rural economy with fewest relatives and clan-members outside of these areas were most vulnerable in 2011.

During the famine, parallel but distinctly different kinds of linkages were invoked to cope with the increasingly difficult circumstances. These linkages function to varying degrees during less fraught times, but were clearly invoked during the famine. They can best be described as three overlapping circles.

The first circle regards immediate kin relations—within the immediate family or among very close relatives. This is where much of the regular remittance activities take place. If a household had connections to someone in the diaspora, or even someone employed in the urban sector who was therefore less subject to the dynamics of the crisis, then that household was likely to survive the famine—regardless of the short-term impacts to their own livelihoods. They could call upon regular outside assistance. On the other hand, in the absence of such linkages—or if the linkages broke down because the remitting individual or household also faced the same set of risks—then connectedness defaulted to the second circle. The first circle is relatively “fixed.”

The second circle is more fluid, and consists broadly of extended family, sub-clan or lineage and community linkages. These linkages overlap with the first circle but also extend well beyond it. Nevertheless, the second circle is also based on “face-to-face” relations—people who actually know and regularly interact with each other (they do *not* have to be in physical proximity given the near ubiquity of telephone-based contact), or at least within social circles that are strongly maintained. This second circle does not necessarily provide a regular source of income or assistance, but in the face of the rapidly worsening conditions in 2011, there was widespread mobilization of this circle; people were called upon to share what they could of their own resources with other members of this circle.

The second circle would be described in contemporary terminology as “community absorptive capacity” and it defines how much of a shock the broader group or network can take before its resource pool collapses. Thus, the “absorptive capacity” of a given household cannot be adequately defined or analyzed without reference to both the first and second circles described here. While the first circle is difficult to measure accurately, the second circle is even more so. It might not be invoked until a crisis hits, and some of the other members of the circle may be affected by the same crisis. Thus, the ability to call on this second circle, and critically, the diversity and depth of resources that flowed in this circle, seemed to be the critical factors in how well a household weathered the famine. While the *structure* of the social network might remain the same in a crisis, the *resource flow* within the network varied, and in some cases declined rapidly.

The extent to which this actually occurred depended on the level of diversification of resources and risk in this second circle. Where most members of the network are in the rural economy—and therefore subject to the same shocks—resources can diminish very quickly. However, where a significant part of the network is located outside the rural economy, the resources within this second circle may be able to mitigate the shock. And the second circle *is* about resource-sharing in some manner or other: it could be about *zakat*; it could be about extending credit (from a shopkeeper or a relative/clan mate) when a household cannot pay cash; and it could be about directly sharing money or other resources. 47

Thus, it is critical to understand who is in and who is out of which circles (even within a clan or lineage-based grouping); the kind of resources circulating in the circle; the extent of these resources; and the diversity of resources and linkages within the “circle” or network. And though it played a critical role in protecting households that did not have regular remittance income, this second circle was *already weakened* by a variety of factors before the famine hit: Al-Shabaab was claiming *zakat* resources for its own use—taking it out of this circle; Some wealthier or better-connected people had moved out of their communities because of Al-Shabaab taxation and harassment, weakening the resource-base. At a certain point in the crisis, this second circle collapsed in certain areas and for certain social groups, and when it did, it collapsed suddenly and left little in the way of a safety net. Some groups, especially those who had more diversified social networks, sought to mobilize higher levels of the clan when the second circle collapsed.

The third circle was much more distant and was comprised of people that one might not know, or where there may not have been a common clan identity. An individual searching to try to find someone—no matter how distant—was heavily reported after about May 2011. This might be an acquaintance, a distant relative, or “big people.” However this might also apply to the highest functional levels of the lineage system. However, this level of the clan/lineage is rarely invoked; it is most commonly sought only in times of large-scale conflict. This third circle is less predicated on face-to-face relations, but rather on more distant claims—usually to a common clan-based identity. This third circle does not function at all in “normal” times, and only in the face of major conflict or crisis in the case of the clan system. Some individuals and some clans were able to find assistance through this circle, but many were not—and those that were excluded fell into famine conditions. This circle came to include a wider sense of Somali solidarity, whereby many diaspora groups were mobilized to contribute to mosques, Somali NGOs, and in some cases invented their own humanitarian projects.

Each of these circles *also* invokes the issues of mobility and diversification, as well as the resources that flow within each of the circles. The second circle clearly exists first and foremost to help individuals or households cope with an idiosyncratic shock—which affect only a few members of the network. A covariate shock—or a combination of covariate shocks such as occurred in 2010–11—eventually undermines the viability of the second circle because the resources that circulate in this circle are finite and cannot be replenished. Analytically, the collapse of the second circle as described here signified the onset of famine conditions in 2011. The collapse of this circle was sudden, and in many cases not easily predictable. But this collapse was indicated by individuals or households beginning to assert claims in the third circle.^48^ This began in May or early June of 2011 (some six to eight weeks before the formal declaration of famine).^49^ If one were to propose an “emic” definition for the declaration of a famine in Somalia, this would almost certainly be it. However, the collapsing of the second level resulted in the mobilization of the third circle, which for some clans averted a collapse into famine. Illustrations of the different experiences of “coping” in 2011 are provided in the following examples.

### 3.4 Differing impacts of social connectedness

The ability to call upon extended family and clan members, located in and outside of the country, in order to mitigate the impact of the worsening crisis, was a recurring theme in the interviews, but the extent to which this was possible varied greatly; clans or social groups with a longer history of migration, education, urbanization and emigration ultimately have more members located outside of the rural economy, and these characteristics are more prevalent for the “noble” clans than for the Rahanweyn and the Bantu/Jarer. There are nevertheless considerable variations in this feature even within the “noble” clans and there are similar hierarchies within the Rahanweyn (as with the Leysan quote).

This phenomenon was most evident in Central regions where (a) the crisis conditions in early 2011 were extremely serious and (b) the mobilization of social networks was intensive and extensive. Respondents from Central regions (and members interviewed in Nairobi and the diaspora) reported how their pastoral kin moved to urban relatives in the small towns of the central regions as the crisis deepened, being absorbed into their extended families (the second circle). However these relatives reportedly could not manage the demands on their resources and called for further help from their Mogadishu and Nairobi-based relatives as well as more distant diaspora relatives. Even respondents in the UK recalled how they organized themselves to raise money when they realized how serious the situation was. A respondent from the UK diaspora stated that:

“*The clan members in Mogadishu responded well. I know one man who donated US $200,000 in one go. Many others were similar in generosity. The members in Mogadishu also contacted the diaspora members of the clan who in three months collected and sent more than US $1 million. The death rate started reducing and the deaths stopped all together before the rains started*”^50^.

In this case, the second circle helped to stem the tide of the crisis, but it was only the third circle that had sufficient resources to contain the deteriorating situation. In contrast, a pastoral clan from Lower Shabelle (the Jiddo, from the Rahanweyn clan), were not able to respond as above. The Jiddo were known as a relatively wealthy sub-clan, owning large numbers of cattle and living in the lower reaches of the Shabelle River, benefiting from the delta formed at its end. In 2011, the river dried up in these lower reaches—an almost unprecedented event that ultimately led to very large numbers of cattle being lost. Many Jiddo become destitute with one respondent described the following:

“*We just kept hoping that it will rain and things will change. Nothing changed and it didn’t rain until the last of my cows died. I had three children then and my wife. We came to Qorioley town. We did not have enough money to pay for the fares to Mogadishu. I had relatives in Qorioley but they were supporting so many other people*”^51^

In this case, a clan with significant cattle wealth did not have enough members or resources outside the rural cattle economy to provide much assistance, and while resources were shared, the rapid depletion of wealth in the second circle (cattle), the situation quickly overwhelmed the ability for mutual assistance. A Jiddo elder in the UK explained it in following way:

*“The Jiddo diaspora community is very small in comparison with other Somali clans. There are 20 in USA, 5 in Australia, 500–1000 in Saudi Arabia, 3 in the UK. In addition the Jiddo do not have business or trading culture and hence do not have big businessmen who could support them in hard times.”*^52^

### 3.5 The limits of social connectedness

While social connectedness is a critical factor in people’s ability to cope with crisis, various nuances influence the effectiveness of that coping. The fact of having social links to relatives in distant locations did not automatically lead to a timely response. Many respondents knew they had distant relatives abroad but were not in regular contact and did not call upon their assistance when times got much harder. In other cases, distant relatives were only fully engaged much later in the crisis and then started to provide financial assistance to help families recover (rather than mitigate the impact of the disaster at the time). In other words, social ties were renewed through the disaster. One respondent explained in the following terms:

“*At first when people were calling me I thought it was the usual calls that I used to receive as people always tell us stories to get money. Then we realized there was a problem later. It took time to mobilize people. It also took time to convince Al-Shabaab to let us help our people. All this contributed to the delay. It is also the case that many nomads put all their efforts in saving their animals and had not much time in soliciting money from relatives until their children were too weak.*”^53^

In other words, even being a member of a wealthy, diversified clan does not guarantee the avoidance of extreme suffering in times of crisis. Those who send frequent financial remittances suffer a form of “fatigue,” and it can take time to organize kin to respond. The strain on those sending money acts to qualify the reification of social ties and North to South remittance flows, that neglect the often precarious financial context of immigrants in their host countries, a condition their own relatives back home often do not fully appreciate.^54^ There is frequently a tension around the trust in or strength of social ties—that people “tell us stories to get money.” This is akin to the notion of “crying wolf” weakening trust in these social networks.^55^

## 4 Social “capital?” or social exclusion and predation?

The hierarchies within Somali society have already been noted, with the Rahanweyn and the Bantu/Jarer occupying a lower status. These two groups were also provided the majority of the displaced populations, as refugees in Ethiopia and Kenya and within Somalia. This was especially the case in Mogadishu, where control of the vast majority of the humanitarian resources, the district commissioners, NGO staff and camp managers, tended to belong to the dominant clan of Mogadishu (the Hawiye). Significant amounts of humanitarian resources were claimed, diverted and/or sold by these various “gate-keepers”—an individual or group who control access to resources—at the expense of extremely impoverished, often starving, people.^56^ The following two quotes, the first from a Somali Bantu person, the second from a Rahanweyn, illustrate the story:

“*We reached Mogadishu. For almost ten days we were depending on begging in the streets with our children because there was no aid*. *It was around late May to early June 2011 that we were taken to one of the Mogadishu IDP camps. They bring food every day but after taking photos the food is taken back from all the people and only 20 percent given to us. Some business people and the owner of the camp, plus the NGO staff are taking the food. We can’t complain because they will chase us from the camp. Sometimes the militia are coming at night taking the few things left and raping girls and women.*”^57^

“*Some days people tell me that some people were given cards to get rations of food but this was done in the night and they gave these cards to their own clans, friends, and acquaintances. I was not a friend or a member of their clan so we were never given a card but we were okay as long as we got something to eat every day. I stayed in these IDP camps for two years during which I saw many tragedies and acts of crime. We were very hungry most of the time. Many children died of malnutrition and diseases such as cholera. I have seen youths from established communities in Mogadishu come into the camps almost every night and rob these IDPs of the few things they had and rape woman with impunity.*”^58^

The exploitation of the famine-displaced reflects not only outright criminality and corruption but also demonstrated the way in which clan identities and power differentiate segments of Somali society and work to exclude and deprive certain groups from access to resources—even to the point of starvation. Menkhaus argued that negotiations with such gatekeepers were as important as those with Al-Shabaab in terms of enabling access, and extremely problematic in both cases.^59^ This control over aid resources by the dominant resident clan also applies in other displaced contexts and was noted, for example, in Dadaab refugee camp in Kenya, during the same study.

In summary, historical processes of marginalization have meant that a long-standing hierarchy within Somali society exists between clan families as well as within them, which today distinguishes those that are more able to contain large-scale (covariate) shocks through their own networked resources from those that are less able to. This underlying vulnerability is exacerbated in times of crisis and humanitarian response, because control of that response is strongly influenced by the more powerful clans (whether individually or collectively).

## 5 Discussion: Famine, coping, and social connectedness

The coping mechanisms outlined in Table 1 would be broadly familiar in any analysis of livelihood activities in a chronically at-risk or crisis-affected area. In the Somalia case, these emphasize diversification and flexibility as well as social connectedness. The cases explored here show that these are characteristics not only of individual or household strategies, but also of social networks. The more flexible or diversified such networks are—in addition to the wealth of network members—the better the chances of individual members’ survival in and recovery from major shocks like the 2011 famine. Diversification here refers more to diversification of risk than to the usual categories of assets and income streams at the level of both the individual or household *and* the network. Table 1 also emphasizes political power and the differential access that groups have to formal assistance—or the capture of aid.

### 5.1 Common themes: social connections and remittances

Much has been written (and speculated about) regarding the role of the diaspora and remittance income in Somalia, and though it has been exhaustively described, it has been stubbornly difficult to quantify at the household level. Questions have lingered about the way in which remittance dynamics change in response to crises. A common theme in individual and household responses was that membership in these networks tends to be stable, and resource flows might remain relatively fixed even in a crisis—described above as the “first circle” of social connectedness. Regular remittance income tended to continue as long as the remitter was not subject to the same hazards as the recipient (hence the importance of diversification of risk within the social network)—and may well increase as the recipient supports more people. But some social dynamics definitely do change in response to worsening conditions (particularly those described here as the “second circle”), and some of these changes may signify the collapse of coping or even the collapse in the ability of existing social networks to support members. In many ways, therefore, this “second circle” as described above is of critical importance to coping and survival. However, some clans were able to effectively mobilize resources within the third circle—at increasingly “distant” levels of the clan or even beyond clan connections.

Thus the factors that strengthen or weaken the second circle—and how these factors may differ among different group—are important to understand. Notions of diversification and flexibility are as important at the level of the social network as they are at the individual or household level—indeed the former goes a long ways towards defining the latter. Under these conditions, it is really not possible to talk about the characteristics and coping capacity of any individual or household—particularly in the context of a major shock—without understanding the characteristics of the social group or network of the individual or household. Some groups were very hard hit by the crisis but were able to cope—or at least survive; others became destitute and/or suffered high levels of mortality

Much was made of how much time the mobilization of an international response took in the face of worsening conditions in the first half of 2011, but these cases also show that organizing an extraordinary response through social networks also took some time—and that to some degree the extraordinary measures described here developed only in response to extraordinary levels of suffering.

Though some superficial similarities may occur, the three “circles” that emerge from the analysis of these interviews from the Somalia famine are *not* the same as the categories of “bonding, bridging, and linking” social capital.” The first two circles could be described as a form of “bonding,” but such a label doesn’t really clarify anything about the dynamics. The third circle can also be understood as a form of bonding, through common identity, but at a distance that is relatively rarely invoked. Transformational changes in communications and transportation—even from the most remote locations in Somalia to the far corners of the globe—have enabled these linkages and probably blurred any analytically useful distinction between “bonding” and “bridging.” In Somalia today, you might be “closer” to someone in the UK or the Middle East than to someone in the next village: face-to-face relations no longer require physical proximity. Examples of individual assisting people from other clans—which might be taken as an example of “bridging”—still relied on face-to-face relationships. This kind of assistance *in extremis*, simply implies that, while “clan” and “sub-clan” are extremely important social categories to understand in Somalia, they do not define the sum total of social relations.

An example of “linking” social connection—not particularly highlighted in this paper, but certainly part of the overall dynamics of the famine and its response—involved Islamic charity networks and provided assistance through mosques or local organizations by linking them to external sources of money or material aid.^60^

More importantly, the relationship between the first and second circles is temporal and related to the ways in which shocks are managed. In the absence of a crisis, the second circle may not be active, or its primary function is to respond to idiosyncratic shocks. It is also maintained in ways other than the flow of money or other resources that affect some members of the network but not all of them. It may expand in unusual ways in the face of extraordinary covariate shocks such as those faced in Somalia in 2011. And for some at least, the second circle ultimately collapsed, triggering widespread stress migration and high levels of human mortality. The third circle was invoked in response to the collapse or severe stressing of the second, and even though it may have involved the search for more distant and better-off connections, it does not correspond directly to the notion of “linking.”

These “circles” define who is in what network and the temporal way in which different networks are invoked. They do not, in themselves, map resource flows or other elements of what might be considered social capital. That requires additional work, and while this is relatively straightforward to describe, it is very difficult to quantify. But it is impossible to understand and differentiate between the coping ability of different groups without understanding these dynamics—even if they are only poorly quantified.

### 5.2 Common themes: social exclusion and predation

Sometimes these relations are as much about exclusion and exploitation as about inclusion and mutual coping. Some groups have been better able to capture external aid just as some groups have a bigger and better educated business community or diaspora—indeed the two often go hand in hand. The issue of gatekeepers and aid “capture” do not fit comfortably within the relatively cheerful categories of “social capital.” Yet clearly people relied on their social connections and identity and their links with clan militias to gain control over aid—sometimes through “aid baiting” or the use of human famine victims to attract humanitarian assistance with the intent to steal or otherwise exploit that aid for their own benefit—or that of their own social networks. Thus social relations and social connectedness can be as much about exclusion as inclusion; as much about victimization and exploitation as about mutual assistance and coping.

### 5.3 Conclusion and implications

Understanding social connectedness is clearly critical for understanding how different groups coped with the famine—and with crisis more generally: how badly people were affected by the same levels of shock, whether they survived, and whether people were able to recover or were left destitute. This connectedness takes place alongside other, better-studied “coping” activities, such as natural resource extraction, asset sales or rationing strategies. But a bigger question lingers about the implications of this analysis for formal policy and practice. In the aftermath of the famine and a similar though somewhat less severe crisis in the Sahel in 2012, much of the policy discourse has focused on the notion of building the resilience of households, communities, and institutions so that they themselves are better able to deal with shocks. The strength of these networks is clearly a major factor that makes households and communities more resilient. Beyond the resilience agenda, another relevant theme is the trend towards localization of humanitarian response and the emphasis on “providers of first resort,” who are almost by definition people who are close by and who may be affected to some degree by the same crisis. Strengthening the capacity of local communities, local government, and local organizations to respond quickly to shocks is increasingly an important part of the discourse about humanitarian action.^61^ The analysis here emphasizes the importance of social connectedness, but whether and how social connectedness can be strengthened by external intervention is not always clear.

It is clearly important to understand these dynamics. But it is equally important to understand that these dynamics have their own logic and rationale—they are not just a local response to external policy. That said, this analysis has a number of important implications for policy and practice—framed here as working hypotheses.

First, many groups were badly affected by the crisis of 2011, but the dynamics analyzed here make it clear that some groups were much better connected and much more able to cope with circumstances. By the same token, however, some groups were much better able to capture formal humanitarian assistance—and indeed this may be part of what enabled them to better withstand the impact of the crisis. So the first working hypothesis is that understanding social connections has obvious implications for the targeting of external humanitarian assistance. However, when targeting has to overcome powerful social dynamics on the ground, experience indicates that it can be extremely difficult to ensure that the most vulnerable groups actually receive the assistance that is allocated to them. External military intervention to ensure that vulnerable groups received assistance backfired dramatically in 1992. No such external intervention was tried in 2011. Indeed in 2011, the external concern about the dynamics of aid capture revolved around the question of whether diverted assistance would fall into the hands of Al-Shabaab. Internally in Somalia, a different set of concerns around aid-capture and constraints to access was highlighted by Menkhaus and related, not to Al-Shabaab, but to gatekeepers or “black cats.” He noted that “Al-Shabaab could be cleared out of much of south Somalia but that would not necessarily guarantee humanitarian access…”^62^

Beyond the issue of targeting is the interpretation of crisis dynamics. As noted, until the time of the famine declaration in July, the situation depicted by the most up to date analysis suggested that the crisis was the worst in central Somalia, but then suddenly famine conditions were found in Bay, Bakool, Lower and Middle Shabelle (which earlier analysis showed to be affected, but not as badly). Much of the speculation since has centered on the question of “what went wrong?” with the analysis of deteriorating conditions in the latter regions. The analysis here suggests a second working hypothesis that the missing element may have been the lack of an understanding of the social connections of affected groups in both areas—that in fact affected groups in the central regions were better connected, with relatively higher proportions of their populations in urban and diaspora locations and more able to cope, even though the conditions might have been as bad or worse there in the early months of 2011 as they were farther to the south. But while such an interpretation is congruent with both the observed current status reports in 2011 and the information on social connectedness as reported here, it is hypothesized—not confirmed—as the explanation.

The persisting question arising is out of this analysis is whether social connectedness can be bolstered through external intervention to improve “community absorptive capacity.” In the aftermath of the crisis, attempts are being made to strengthen local early warning, and to build community contingency funds, capitalized by joint investment from external and community sources. These efforts go beyond simply improving livelihoods assets or diversifying income streams, and are attempts to strengthen the role of “responders of first resort.” For the most part it is too early to judge the success of these interventions. However, engaging in complex, contested social contexts is extremely difficult, especially for humanitarian actors whose focus is typically on delivery. So a third working hypothesis is understanding these dynamics is critical, but not something that one does overnight or in the context of “rapid assessment.” The analysis here has important implications about mechanisms to ensure equal access to such resources, and also for the targeting of interventions to improve these capacities. For the most part, however, targeting of interventions in the post-famine period is still being managed in such a way as to minimize the likelihood that Al-Shabaab might benefit from diverted aid. In other words, aid is being allocated according to risk criteria, not need—so in fact many of the areas most affected by the famine are *not* included in these interventions.

On the other hand, while proactively intervening on the basis of the analysis here may not be straightforward, it is quite clear that any attempt to bolster resilience in Somalia should seek to avoid undermining the very strategies on which Somali communities rely. So the 4th working hypothesis is that attempts to isolate Somali money transfer companies from international banking systems (again, in the name of counter-terrorism) undermine the resilience of Somali communities where the international transfer of money is an important part of daily life and the response to crisis conditions.^63^ It makes no sense for external actors to try to “build resilience” while at the same time self-evidently undermining it.

Finally, measuring social connectedness presents substantial methodological problems. The analysis here was based on two years of fieldwork, mostly conducted retrospectively after the famine had ended. But this analysis suggests that the way social networks function *in extremis* is qualitatively different from the way they function under more “normal” circumstances—and the monitoring of these changes could be a very important kind of information for early warning and response. Certainly understanding and predicting the collapse of networks of support is critical. Monitoring the kinds of information presented here is possible but require different monitoring protocols than those currently used. And framing social connectedness in terms of clan or social identity is highly political, and an area that most conventional information systems avoid. Solid knowledge about livelihood patterns and geographic locations of different groups can help to depoliticize the analysis, but in situation of rapid population displacement, these can shift quickly, whereas social identity remains constant.

Nevertheless, understanding the capacity of households and communities to cope with deteriorating circumstances is critical to both analysis of and intervention in future crises. This analysis has highlighted both the necessity and the complexity of understanding social connectedness in the Somalia context. While understanding social identity in all its complexity is critical, to a large degree during the famine, the social category that most defined the ability to survive was described in terms of lineage and clan, but under extreme circumstances may go beyond these categories. The extent to which these connections can be understood and mapped will provide a deeper understanding of resilience in Somalia, and therefore an important component of preventing future famines.

## Figures and Tables

**Table 1:** Typology of resilience and coping in the Somali famine.

Category	Examples	Level	Application/severity
Diversification	• Diversify livelihoods and assets • Diversification of risk • Diversify against drought risk (riverine farming and/or camels) • Have a foot in the urban economy	Individual/household Some diversification within clan or larger group	Mostly applies in the longer term and a means of reducing risk, not as a means of coping with shocks
Flexibility	• Physical mobility with livestock • Labor mobility (employment) • Exploit different opportunities (including humanitarian aid) • Outmigration as a last resort	Household Community-level decisions about when to move?	Limited ability to move condemned some small-scale livestock holders, but others suffered large losses far from home
Social “connectedness”	• Forms of mutual support • Usual: remittances; unusual: diaspora or urban contacts, etc. • Having “someone to cry to”; three overlapping circles model	“Second circle” community level/clan level Partly business level	Diaspora remittances stepped up in famine: food, water trucking Third circle as “system failure”
Political power	• Access to/control over aid	Household Community	Gatekeepers from powerful clans in IDP settings
Crisis asset protection	• Sharing food or assets with livestock • Buying water for livestock • Moving livestock in search of grazing and water • Leaving someone behind to protect land if migrating • Decision making about when to sell animals, when to move, etc.	Household Community	Feeding cattle thatch from roofs during drought Timing of livestock sales Out-migration usually as a last resort
Asset sales or depletion	• Sale of livestock • Sale of other productive assets • Land pledging or mortgaging	Household Community	
Rapid livelihood adaptation	• Renting farmland (esp. riverine) to protect animals (access water/fodder) • Sharing lactating animals—move with non-lactating animals • Natural resource extraction: firewood, charcoal, thatch grass • Search for casual wage employment	Household or inter-household Wage labor in community as form of social reciprocity albeit a form of exchange	Some of these are “normal” livelihoods for poor people, others are coping strategies in crisis
Credit	• Use of savings/borrowing/debt • Borrowing/purchase on credit as one form of social connectedness	Household Business	Social networks portrayed in positive light; can lead to long-term indebtedness
Consumption strategies	• Changing diets • Borrowing food or money • Rationing strategies • Going hungry		
Household and inter-household demographic strategies	• Family splitting—both consumption- minimization strategy and resource-acquisition maximization strategy • Opportunistic access to aid resources/household splitting • Labor-sharing	Household Inter-household/community	

Data: Field Interviews 2012–14.

## Footnotes

1 Although the literature relies heavily on the term “social capital,” for a variety of reasons we prefer to analyze social networks and social connectedness. For the sake of clarity, we refer to the literature by its own chosen name, but the evidence here tends to reject the notion that the sum total of complex social relations can be boiled down to some kind of “capital.”

2 Other interviews included people in refugee hosting communities in Kenya and Ethiopia, as well as government and aid agency officials, which were not analyzed here, except for general background.

3 A selection of these narratives have been compiled and briefly analyzed in a separate and much longer paper. See Majid et al. “Narratives of Famine – Somalia 2011.” (Medford: Tufts University, 2016).

4 For example, in Luuq, we spoke to Marehan, Dir and Gabaweyne identity groups and in Qorioley to Dubane/Jarer, Garre and Jiddo.

5 Jaber Gubrium et al., *The Sage Handbook of Interview Research: The Complexity of the Craft* (New York: Sage, 2012).

6 Virginia Luling. “Genealogy as Theory, genealogy as tool: aspects of Somali ‘Clanship’.” *Social Identities*, 12(4): 471–485.

7 Roland Marchal. “Warlordism and Terrorism: How to Obscure an Already Confusing Crisis? The Case of Somalia.” *International Affairs,* 83 no 6 (2016): p. 1098.

8 See I.M. Lewis, *A Pastoral Democracy: A Study of Pastoralism and Politics Among the Northern Somali of the Horn of Africa*, London: James Currey (1961). Also see Cindy Horst, *Transnational Nomads: How Somalis Cope with Refugee Life in the Dadaab Camps of Kenya*, New York and Oxford: Berghahn Books (2006)

9 See Daniel Maxwell and Nisar Majid, *Famine in Somalia: Competing Imperatives, Collective Failures, 2011–2012.* (London and New York: Hurst Publishers/Oxford University Press, 2016).

10 Francesco Checci and W. Courtland Robinson, “Mortality among Populations of Southern and Central Somalia Affected by Severe Food Insecurity and Famine During 2010–2012,” (Rome/Washington, D.C.: FAO/FEWS NET, 2013).

11 For a detailed analysis of the famine as a whole, see Daniel Maxwell and Nisar Majid, *Famine in Somalia: Competing Imperatives, Collective Failures, 2012–2012* (London: Hurst Publishers, 2016).

12 Checci and Robinson, “Mortality among Populations of Southern and Central Somalia Affected by Severe Food Insecurity and Famine During 2010–2012.”

13 Numerous factors related to the course of the crisis, including a detailed analysis of the causal and complicating factors; the “early warning/late response” phenomenon; the role of Al-Shabaab and counter-terrorism restrictions in constraining mitigation and response and numerous other issues are outlined in Maxwell and Majid, *Famine in Somalia: Competing Imperatives, Collective Failures, 2012–2012*.

14 The study also included focused research on the role and response of Islamic actors (both donors and charities) but while many of these agencies responded to the famine, we treated this as a different kind of intervention. See Daniel Maxwell and Nisar Majid, *Famine in Somalia: Competing Imperatives, Collective Failures, 2011*–*12* (New York: Oxford University Press, 2016).

15 Amartya Sen, *Poverty and Famines: An Essay on Entitlement and Deprivation* (New York: Oxford University press, 1981).

16 Jane Corbett, “Famine and Household Coping Strategies,” *World Development* 16, no. 9 (1988). Susanna Davies, *Adaptable Livelihoods. Coping with Food Insecurity in the Malian Sahel* (London: MacMillan Press, 1996).

17 Michael J. Watts, *Silent Violence: Food, Famine and Peasantry in Northern Nigeria* (Berkley: University of California Press, 1983).

18 F. Ellis, Stephen Devereux, and P. White, *Social Protection in Africa* (Cheltenham, UK; Northampton, MA, USA: Edward Elgar, 2009); Frank Ellis, “Household Strategies and Rural Livelihood Diversification,” *The Journal of Development Studies* 35, no. 1 (1998); Christopher McDowell and Arjan de Haan, “Migration and Sustainable Livelihoods: A Critical Review of the Literature,” (Brighton: IDS, 1997).

19 “Migration and Sustainable Livelihoods: A Critical Review of the Literature.”

20 See Jonathan Xavier Inda and Renato Rosaldo, *The Anthropology of Globalization. A Reader*. (Blackwell Publishing, 2002: 19).

21 Anna Lindley, “The Early Morning Phone Call: Remittances from a Refugee Diaspora Perspective: Working Paper No. 47,” (Oxford: Centre on Migration, Policy and Society, 2007); McDowell and de Haan, “Migration and Sustainable Livelihoods: A Critical Review of the Literature”; R. McLeman and B. Smit, “Migration as an Adaptation to Climate Change,” *Climatic Change* 76, no. Journal Article (2006); Laura Hammond, “Family Ties.” Remittances and Livelihood Support in Puntland and Somaliland,” (Nairobi, Kenya: FSNAU, 2013).

22 Anna Wetterberg, “Crisis, Social Ties, and Household Welfare: Testing Social Capital Theory with Evidence from Indonesia,” (Washington DC: World Bank, 2005).

23 Pierre Bourdieu, “The Forms of Capital. In J. Richardson (Ed.) (New York, Greenwood), 241–258.,” in *Handbook of Theory and Research for the Sociology of Education*, ed. J. Richardson (New York: Greenwood, 986). p. 248.

24 James Coleman, “Social Capital in the Creation of Humanitarian Capital. 94, Suppl. Organizations and Institutions: Sociological and Economic Approaches to the Analysis of Social Structure, S95-S120,” *American Journal of Sociology* 94, no. Supplement (1988). P. S98.

25 Robert Putnam, *Making Democracy Work: Civic Traditions in Modern Italy* (Princeton, NJ: Princeton University Press, 1993). (Putnam, 1993, p.167).

26 Daniel Aldrich, *Building Resilience: Social Capital in Post-Disaster Recovery* (Chicago: University of Chicago Press, 2012).

27 Robert Putnam, *Bowling Alone: The Collapse and Revival of American Community* (New York: Simon and Schuster, 2000).

28 Aldrich, *Building Resilience: Social Capital in Post-Disaster Recovery*.

29 Ibid.

30 Simon Szreter and Michael Woolcock, “Health by Association? Social Capital, Social Theory, and the Political Economy of Public Health,” *International Journal of Epidemiology* 33, no. 4 (2004). p. 655.

31 Aldrich, *Building Resilience: Social Capital in Post-Disaster Recovery*. p. 33.

32 Quinn Bernier and Ruth Meinzen-Dick, “Resilience and Social Capital” in *2020 Conference Paper 4* (Washington DC: IFPRI, 2014).

33 W. Neil Adger, “Social Capital, Collective Action, and Adaptation to Climate Change,” *Economic Geography* 79, no. 387–404 (2003).

34 Bernier and Meinzen-Dick, “Resilience and Social Capital”.

35 Peggy Levitt and B. Nadya Jaworski. “Transnational Migration Studies: Past Developments and Future Trends.” Annual Review of Sociology, 33: 129–156. (2007), p. 137.

36 Lee Cassanelli, “Speculations on the Historical Origins of the ‘Total Somali Genealogy’,” in *Milk and Peace, Drought and War: Somali Culture, Society and Politics*, ed. Markus Hoehne and Virginia Luling (London: Hurst, 2009).

37 Gardner and El Bushra, *Somalia - the Untold Story*.

38 Alex de Waal, *Famine Crimes: Politics and the Disaster Relief Industry in Africa*, African Issues (London: James Currey, 1997).

39 UNDP, “Human Development Report: Somalia” (Nairobi/New York: UN Development Programme, 2001).

40 Corbett, “Famine and Household Coping Strategies.” Davies, *Adaptable Livelihoods. Coping with Food Insecurity in the Malian Sahel*.

41 The resilience literature in general, and on Somalia has grown rapidly since the famine. The most important conceptual work on this is, Christopher B. Barrett and Mark A. Constas, “Toward a Theory of Resilience for International Development Applications” *Proceedings of the National Academy of Sciences* 111, no. 40 (2014). In terms of programmatic applications, see, Tim Frankenberger et al., “Community Resilience: Conceptual Framework and Measurement Feed the Future Learning Agenda,” (Rockville, MD: USAID Feed the Future FEEDBACK Project, 2013).

42 Judith Gardner and Judith El Bushra, *Somalia - the Untold Story*, ed. Judith Gardner and Judith El Bushra (London: CIIR and Pluto Press, 2004).

43 See Anna Lindley, “Migrant Remittances in the Context of Crisis in Somalia: A case-study of Hargeisa.” Background Paper (London: Humanitarian Policy Group, Overseas Development Institute, 2006).

44 For example, see Jane Corbett, “Famine and Household Coping Strategies.”

45 The Leysan are one of the most pastoral and wealthy sub-clans of the Rahanweyn with a small business community and a small but significant diaspora population.

46 Field Interview. 2013. See Interview 02-003, Majid et al., 2016

47 Field interviews. 2013–14. See Majid et al., 2016 (for this point shall we just reference the whole other paper?

48 One of our senior researchers described this phenomenon (in his own case) in terms of beginning to receive phone calls from people he didn’t know, asking for assistance. This began in about May 2011.

49 Field interviews. 2013–14; see Majid et al., 2016 for a selection.

50 Field Interview. 2013. See Interview 01-005, Majid et al., 2016.

51 Field Interview. 2012.

52 It is not only having a diaspora, but where they are located and in what employment status that is important; the Saudi Arabian diaspora mentioned may not be able to raise the same funds as an equivalent number in Europe or the USA for example. See Interview 04-005, Majid et al., 2016.

53 Field Interview. 2014. See Interview 01-005, Majid et al., 2016.

54 Lindley, “The Early Morning Phone Call: Remittances from a Refugee Diaspora Perspective.” Working Paper No. 47.

55 Nisar Majid, “Livelihoods, Development and the Somali Diaspora” (University of Bristol, 2013).

56 Human Rights Watch, “Hostages of the Gatekeepers: Abuses against Internally Displaced in Mogadishu, Somalia,” (New York: Human Rights Watch, 2013).

57 Field Interview. 2014 Interview 02-004, Majid et al., 2016.

58 Field Interview. 2013. Interview 04-002, Majid et al., 2016.

59 Ken Menkhaus, “No Access: Critical Bottlenecks in the 2011 Somali Famine,” *Global Food Security* 1, no. 1 (2012).

60 For details on this, see Maxwell and Majid, *Famine in Somalia: Competing Imperatives, Collective Failures, 2012–2012*. Respondents from these actors claimed that, for the most part, they did not receive additional resources in 2011 until the declaration of the famine and the global media coverage, in spite of trying to do so earlier in recognition of the developing crisis.

61 See for example, much of the preparatory analysis for the World Humanitarian Summit to be held in Istanbul in May 2016.

62 Menkhaus, “No Access: Critical Bottlenecks in the 2011 Somali Famine.” p. 34.

63 The most flagrant example of this was the pressure from the UK government for Barclay’s Bank to close the account of Dahabshiil (the largest of the money transfer companies) at the same that it was investing millions of Pounds Sterling in “resilience” programming in Somalia. BBC 2013: http://www.bbc.com/news/business-23030943
